# Effects of Snake Fruit–Infused Massage Oil With Traditional Thai Massage on Skin Quality: A Randomized Controlled Trial

**DOI:** 10.1155/tswj/2446967

**Published:** 2026-04-07

**Authors:** Suwipa Intakhiao, Warumpa Suwannarat, Thirapit Subongkot, Thanchanok Sirirak, Phannapat Piyaneeranat, Sukrisd Koowattanatianchai, Piyapong Prasertsri

**Affiliations:** ^1^ Faculty of Allied Health Sciences, Burapha University, Chonburi, Thailand, buu.ac.th; ^2^ Faculty of Pharmaceutical Sciences, Burapha University, Chonburi, Thailand, buu.ac.th; ^3^ The Research Unit in Synthetic Compounds and Synthetic Analogues from Natural Product for Drug Discovery (RSND), Burapha University, Chonburi, Thailand, buu.ac.th; ^4^ Division of Cardiology, Department of Medicine, Burapha Hospital, Burapha University, Chonburi, Thailand, buu.ac.th

**Keywords:** body oil, massage, skin health, snake fruit, spa

## Abstract

**Background:**

*Salacca zalacca* (snake fruit) is rich in antioxidants, polyphenols, organic acids, and vitamin C. This study aimed to evaluate the effectiveness of body massage oil containing snake fruit extract, in conjunction with traditional Thai massage (TTM), on skin quality in healthy individuals.

**Methods:**

Seventy‐one participants aged 18–35 years were randomly assigned to one of three groups: (1) control group (*n* = 23) receiving TTM without oil; (2) Treatment‐1 group (*n* = 23) receiving TTM with pure coconut oil; and (3) Treatment‐2 group (*n* = 25) receiving TTM with snake fruit extract–infused oil. All participants received 60‐min massages once weekly for 12 weeks. Skin parameters including elasticity, moisture, melanin, and oiliness were assessed at the neck, back, arm, and leg regions.

**Results:**

After 12 weeks, skin elasticity significantly improved at all assessed regions in all groups (*p* < 0.001), with no significant between‐group differences. Skin melanin levels significantly decreased at the back and leg regions across all groups (*p* < 0.05), with no between‐group differences observed. Skin moisture significantly increased at the leg region only in the Treatment‐2 group (*p* = 0.003). Skin oiliness significantly increased at all measured regions in both oil‐based groups (Treatment‐1 and Treatment‐2) (*p* < 0.05) and was significantly higher than in the control group (*p* < 0.05), except at the back region in the Treatment‐2 group.

**Conclusion:**

Massage oil containing snake fruit extract demonstrated specific benefits in enhancing skin oiliness and localized moisture. However, it did not confer overall superiority over conventional coconut oil, while improvements in elasticity and melanin appeared to be primarily attributable to the massage technique itself.

**Trial Registration:** ClinicalTrials.gov Identifier: NCT06227260

## 1. Introduction

*Salacca zalacca*, commonly known as snake fruit, is a tropical fruit cultivated in several Southeast Asian countries, including Thailand. It is a rich source of bioactive compounds such as antioxidants, polyphenols, organic acids, and vitamin C. Notably, snake fruit contains a variety of phenolic compounds, including chlorogenic acid, epicatechin, isoquercetin, neochlorogenic acid, ferulic acid, gallic acid, procyanidin B2, syringic acid, and caffeic acid [1]. These compounds exhibit potent antioxidant activity and play a critical role in neutralizing free radicals that can otherwise damage cellular components [2]. Furthermore, the polyphenol and antioxidant contents of snake fruit pulp have been reported to be higher than those found in mango and mangosteen, and comparable to levels observed in kiwifruit [3].

In the field of nutricosmetics, research has primarily focused on bioactive compounds with the ability to inhibit skin‐aging enzymes, as well as those possessing antimicrobial, antioxidant, and anti‐inflammatory properties, all of which contribute to improved skin health and protective effects against environmental stressors [4]. Within this context, snake fruit extract has demonstrated both antioxidant activity and tyrosinase inhibition—an enzyme critical to melanin biosynthesis and thus central to the regulation of skin pigmentation [5]. Notably, a study using ethanolic extract of snake fruit reported that a topical cream containing 3% extract significantly reduced the skin melanin index, supporting its potential application in skin‐lightening formulations [6].

Massage therapy is increasingly incorporated into traditional and integrative medical practices and has been studied in relation to various health conditions, including stress, infertility, prematurity, full‐term newborn care, autism, dermatological issues, pain syndromes, hypertension, autoimmune diseases, immune dysfunctions, and age‐related conditions [7]. Among the different types of massages, Swedish massage and traditional Thai massage (TTM) are commonly investigated [7]. The use of massage oils—particularly in aromatherapy massage—is a common adjunct to enhance the therapeutic effects of manual techniques [8]. In line with this, numerous plant‐derived oils such as olive oil, olive pomace oil, sunflower seed oil, coconut oil, safflower seed oil, argan oil, soybean oil, peanut oil, sesame oil, avocado oil, borage oil, jojoba oil, oat oil, pomegranate seed oil, almond oil, bitter apricot oil, rosehip oil, German chamomile oil, and shea butter have been utilized in topical skin applications due to their anti‐inflammatory and antioxidant properties. These oils are known to promote wound healing, support skin barrier repair, and improve overall skin health [9].

TTM combines deep tissue pressure, acupressure, and assisted stretching, producing mechanical stimulation that enhances circulation, lymphatic drainage, and dermal microperfusion. These effects may increase the penetration and bioavailability of topically applied bioactive compounds. When combined with snake fruit extract—rich in phenolic antioxidants—TTM‐induced improvements in vascular dynamics and tissue relaxation may facilitate deeper diffusion into the skin [10]. Additionally, massage‐related increases in circulation and hydration may synergistically enhance nutrient delivery, waste removal, and skin elasticity, further supporting skin health [11].

Accordingly, this study developed a body massage oil infused with snake fruit extract and evaluated its effects in combination with TTM on body skin quality. This approach extends the application of snake fruit beyond nutritional use and highlights its potential as a natural, bioactive ingredient in health and wellness products. The findings may inform the development of functional spa and cosmeceutical formulations that integrate traditional massage techniques with plant‐based bioactive compounds.

## 2. Materials and Methods

### 2.1. Participants and Sample Size Calculation

This study was conducted as a randomized controlled trial. A total of 75 healthy male and female volunteers aged 18–35 years who expressed interest in TTM were enrolled in Chonburi Province, Thailand. Participants were excluded if they met any of the following criteria: (1) history of adverse reactions to massage; (2) history of allergic skin conditions; (3) presence of abnormal skin conditions on the body; (4) open wounds or skin lesions; or (5) known allergies to coconut oil or other plant‐based oils.

Based on standardized effect size conventions proposed by Cohen [12] and effect magnitudes reported in previous clinical studies examining changes in skin hydration following topical interventions [13], a moderate‐to‐large standardized effect size was assumed (*f* = 0.40; equivalent to Cohen’s d ∼0.75). Sample size estimation was performed using a one‐way analysis of variance (ANOVA) with three groups, a significance level of *α* = 0.05, and a statistical power of 0.80, implemented in G∗Power software (Version 3.1.9.4). This analysis indicated that a minimum of 22 participants per group was required. To accommodate an anticipated dropout rate of approximately 20%–25%, the target sample size was increased to 25 participants per group, resulting in a total sample size of 75 participants.

### 2.2. Ethical Considerations

All eligible participants provided written informed consent prior to undergoing any screening procedures. They were thoroughly informed—both verbally and in writing—about the study’s objectives, experimental procedures, and potential risks and benefits, as well as their rights and responsibilities as research participants. The study protocol was approved by the Human Ethics Committee of Burapha University (Approval No. IRB1‐028/2566, approval date: March 20, 2023) and by the Biosafety Committee for Research at Burapha University (Approval No. 6/2566, approval date: April 20, 2023).

### 2.3. Participant Screening and Safety Assessment

Eligible participants were screened using health questionnaire forms, vital signs assessment, and body composition analysis. The health questionnaire included items on a COVID‐19 symptom checklist, general demographic information, medical history, exercise participation, and conditions potentially affecting massage tolerance—with or without oil application—such as muscle soreness, dermatologic issues, allergic reactions, fever, and signs of inflammation. Blood pressure and heart rate were measured using an automatic blood pressure monitor (Omron HEM‐7121; OMRON Healthcare, Inc., Kyoto, Japan). Body temperature was assessed with a digital clinical thermometer (Terumo C205; Terumo Corporation, Tokyo, Japan). Body composition was evaluated using a bioelectrical impedance body composition analyzer (InBody270; InBody Co., Ltd., Daejeon, Korea).

Additionally, all participants were asked to apply both coconut oil and snake fruit extract–infused oil during the screening process to assess for any potential allergic reactions or abnormal skin responses prior to randomization.

### 2.4. Preparation of Snake Fruit Extract and Massage Oil Formulation

Fresh snake fruit pulp was freeze‐dried and extracted with hexane at a sample‐to‐solvent ratio of 1:20 (w/v). The mixture was homogenized by sonication for 30 min and subsequently filtered through cotton. The extraction was repeated twice to maximize the yield. The combined filtrates were concentrated under reduced pressure using a rotary evaporator to remove the solvent. The resulting extract yielded 0.02 g of concentrated extract per 0.11 g of fresh snake fruit pulp (yield = 20.20%).

For the massage oil formulation, 308 mg of the concentrated snake fruit extract was dissolved in 22 g of caprylic/capric triglyceride, yielding a concentration of 1.40% (w/w). This solution was incorporated into the base oil blend at 0.20% (w/w), resulting in a final crude extract concentration of 0.0028% (w/w) in the massage oil formulation. The selected extract concentration was based on levels commonly used in topical cosmetic formulations and on previous reports demonstrating antioxidant and tyrosinase‐inhibitory activities of snake fruit extracts. All extraction and formulation procedures were conducted at the Faculty of Pharmaceutical Sciences, Burapha University, by two trained researchers (T.S. and T.S.), in a laboratory accredited under the Peer Evaluation Standard of the National Research Council of Thailand.

Pure coconut oil used in this study was supplied by Coco‐House Natural Product Co., Ltd. (Pathum Thani, Thailand). This product is a high‐quality, commercially available coconut oil commonly used in spa and massage settings. The massage oils used in this study differed in their base oil composition. Coconut oil represents a single‐ingredient base oil, reflecting standard practice in traditional massage and spa applications. In contrast, the snake fruit extract–infused massage oil was a formulated product in which the extract was incorporated as an active ingredient into a blended oil base consisting primarily of mineral oil, C11–12 isoparaffin, isopropyl myristate, and caprylic/capric triglyceride, together with supportive cosmetic excipients. Accordingly, the snake fruit extract–infused massage oil represents a finished, multifunctional formulation rather than an extract‐only or base oil–matched intervention.

### 2.5. Study Design, Randomization, and Intervention

Upon arrival at the research facility, participants first underwent baseline skin quality assessments. They were then randomly assigned to one of three groups: control (*n* = 25), Treatment‐1 (*n* = 25), or Treatment‐2 (*n* = 25). Randomization was performed using simple random allocation generated by IBM SPSS Statistics software (Version 25), with allocation concealment ensured through central randomization. A principal investigator (S.I.) generated the random sequence and assigned participants to their respective groups.

Participants in the control group received TTM without oil and were instructed to refrain from using body oils or exfoliating products throughout the 12‐week study period. Participants in the Treatment‐1 group received TTM combined with pure coconut oil, while those in the Treatment‐2 group received TTM combined with snake fruit extract–infused massage oil. All massage sessions were delivered by licensed Thai traditional medicine practitioners to ensure procedural consistency. Each session lasted 60 min and was conducted once weekly for 12 weeks.

Due to the nature of the intervention, participant blinding was not feasible. However, assessor blinding was implemented to minimize bias. Skin quality assessments at both baseline and postintervention were performed by a trained assessor (W.S.) who was blinded to group allocation throughout the study. Participant coordination and data collection logistics were managed by researcher P.P., who did not participate in outcome assessments. Data analysis was conducted independently by another researcher (P.P.), who remained blinded to intervention assignments.

Following completion of the 12‐week intervention, all participants underwent postintervention skin quality assessments. Participants were withdrawn from the study if they attended fewer than 80% of the scheduled massage sessions or withdrew voluntarily at any time. To control for external influences, participants were instructed to refrain from showering for at least 4 h prior to skin measurements. All assessments were conducted under standardized environmental conditions (room temperature 25°C and relative humidity 40%–50%).

### 2.6. Skin Quality Assessment

Participants’ skin quality—including elasticity, moisture, melanin, and oiliness—was assessed at both the left and right sides of the neck, lower back, forearms, and legs while participants were in the prone position. Values from both sides were summed, averaged, and reported for each anatomical region. All measurements were conducted using the Dual Cutometer Multiprobe Adapter System (MPA 580; Courage + Khazaka electronic GmbH, Cologne, Germany). Specific probes used for the assessments included skin elasticity (Cutometer 580 probe), skin moisture (Corneometer CM825 probe), skin melanin and erythema (to assess potential skin irritation) (Mexameter MX18 probe), and skin oiliness (sebum levels) (Sebumeter SM815 probe). All values were reported in arbitrary units (a.u.), as per the standard method described by Suwannarat et al. [14].

### 2.7. Data Analyses

All data were analyzed using SPSS Statistics software Version 25 (IBM Corp., Armonk, NY, USA) and are presented as mean ± standard deviation (SD). The normality and homogeneity of variance were assessed using the Shapiro–Wilk test and Levene test, respectively. Between‐group differences before and after the intervention were analyzed using one‐way ANOVA, followed by the Bonferroni post hoc test for multiple comparisons. Within‐group differences (pre‐ vs. postintervention) were analyzed using the paired *t*‐test. A *p* value of < 0.05 was considered statistically significant.

## 3. Results

### 3.1. Baseline Participant Characteristics

Participants were recruited between May 9, 2023, and August 15, 2023. Follow‐up assessments were conducted from August 16, 2023, to November 22, 2023, with the final participant visit occurring on November 22, 2023. Of the 75 participants initially enrolled, four participants (5%) did not complete the postintervention assessments due to discomfort. Thus, data from 71 participants (95%) were included in the final analysis. The final sample consisted of 22 males (31%) and 49 females (69%), with a mean age of 23.99 ± 5.68 years. Group distribution was as follows: control group (*n* = 23), Treatment‐1 group (TTM with coconut oil; *n* = 23), and Treatment‐2 group (TTM with snake fruit extract–infused oil; *n* = 25).

**FIGURE 1:**
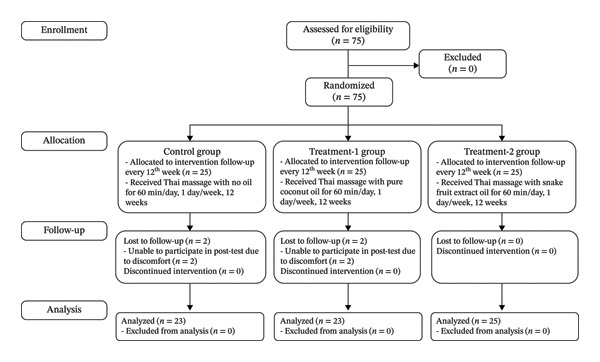
CONSORT flow diagram.

There were no statistically significant differences among the three groups with respect to sex, age, height, body mass, body mass index, or other baseline characteristics (Table 1), indicating comparability across groups at the start of the intervention. Additionally, no serious adverse events were reported by any participants related to the use of either pure coconut oil or snake fruit extract–infused oil throughout the 12‐week study period. The participant flow from enrollment to analysis is summarized in the CONSORT flow diagram (Figure 1).

**TABLE 1:** Baseline physical characteristics of participants prior to the 12‐week interventions.

Characteristics	Control group (*n* = 23)	Treatment‐1 group (*n* = 23)	Treatment‐2 group (*n* = 25)
Age (yrs)	24.62 ± 5.68	24.25 ± 5.72	25.83 ± 8.99
Sex (male/female) (%)	8/15 (35/65)	6/17 (26/74)	8/17 (32/68)
Height (m)	1.65 ± 0.09	1.61 ± 0.10	1.64 ± 0.09
Body mass (kg)	61.70 ± 13.52	63.01 ± 15.16	60.78 ± 15.14
Body mass index (kg/m^2^)	22.66 ± 4.52	23.21 ± 7.07	22.45 ± 4.45
Body fat (%)	27.16 ± 8.50	29.67 ± 9.21	27.06 ± 7.31
Muscle mass (kg)	42.63 ± 8.58	41.28 ± 9.42	41.83 ± 10.54
Water mass (kg)	50.29 ± 4.49	49.23 ± 4.38	50.63 ± 3.61
Visceral fat level	5.12 ± 3.55	5.78 ± 3.59	4.90 ± 3.73
Basal metabolic rate (kcal/day)	1363.08 ± 242.98	1336.43 ± 248.88	1354.46 ± 308.50

*Note:* Data are mean ± SD, frequencies, percentages.

### 3.2. Skin Elasticity

**FIGURE 2:**
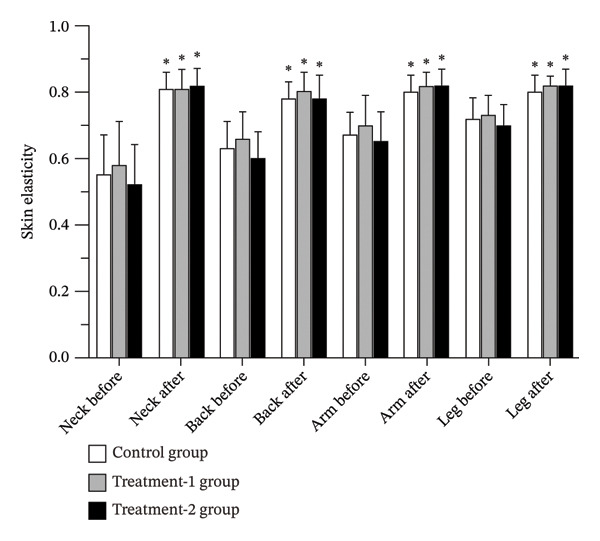
Skin elasticity levels of participants before and after the 12‐week interventions.

After the 12‐week intervention, skin elasticity significantly increased at the neck, back, arm, and leg regions in all groups, including the control (*p* < 0.001), Treatment‐1 (*p* < 0.001), and Treatment‐2 (*p* < 0.001) groups. However, there were no statistically significant differences in skin elasticity among the three groups at any of the measured regions (Figure 2 and Table 2).

**TABLE 2:** Skin elasticity, moisture, melanin, oiliness, and erythema levels of participants before and after the 12‐week interventions.

Parameters/Regions	Control group (*n* = 23)		Treatment‐1 group (*n* = 23)			Treatment‐2 group (*n* = 25)		95% CI	Effect size (partial *η* ^2^)
	Before	After	Before		After	Before	After		
Skin elasticity									
Neck (a.u.)	0.55 ± 0.12	0.81 ± 0.05^∗^	0.58 ± 0.13		0.81 ± 0.06^∗^	0.52 0.12	0.82 ± 0.05^∗^	−0.06, 0.03^a^ −0.04, 0.04^b^ −0.03, 0.06^c^	0.018
Back (a.u.)	0.63 ± 0.08	0.78 ± 0.05^∗^	0.66 ± 0.08		0.80 ± 0.06^∗^	0.60 ± 0.08	0.78 ± 0.07^∗^	−0.05, 0.04^a^ −0.06, 0.03^b^ −0.05, 0.03^c^	0.011
Arm (a.u.)	0.67 ± 0.07	0.80 ± 0.05^∗^	0.70 ± 0.09		0.82 ± 0.04^∗^	0.65 ± 0.09	0.82 ± 0.05^∗^	−0.05, 0.02^a^ −0.05, 0.02^b^ −0.04, 0.03^c^	0.026
Leg (a.u.)	0.72 ± 0.06	0.80 ± 0.05^∗^	0.73 ± 0.06		0.82 ± 0.03^∗^	0.70 ± 0.06	0.82 ± 0.05^∗^	−0.05, 0.01^a^ −0.05, 0.01^b^ −0.03, 0.03^c^	0.053

Skin moisture									
Neck (a.u.)	58.18 ± 14.83	58.14 ± 18.36	67.60 ± 16.51		65.84 ± 17.47	65.14 ± 14.26	70.97 ± 20.17	−20.68, 7.87^a^ −25.49, 2.13^b^ −18.71, 8.17^c^	0.061
Back (a.u.)	55.96 ± 12.74	61.92 ± 21.87	57.81 ± 16.31		59.80 ± 17.64	57.11 ± 12.79	64.50 ± 17.78	−11.56, 16.86^a^ −15.95, 11.89^b^ −18.43, 9.07^c^	0.010
Arm (a.u.)	56.89 ± 23.02	51.68 ± 16.13	55.26 ± 10.26		57.36 ± 13.07	54.18 ± 10.92	60.74 ± 14.39	−16.17, 5.46^a^ −19.39, 1.85^b^ −13.89, 7.05^c^	0.059
Leg (a.u.)	41.26 ± 9.08	43.71 ± 16.06	45.78 ± 10.94		49.60 ± 10.53	41.81 ± 9.94	51.84 ± 13.15^∗^	−14.15, 5.64^a^ −17.18, 1.91^b^ −12.94, 6.18^c^	0.055

Skin melanin									
Neck (a.u.)	362.73 ± 99.47	336.76 ± 135.87		378.48 ± 104.98	278.97 ± 119.13^∗^	370.52 ± 106.89	287.09 ± 100.27^∗^	−4.59, 140.09^a^ −16.18, 125.37^b^ −83.93, 57.62^c^	0.082
Back (a.u.)	208.60 ± 77.50	181.60 ± 73.65^∗^		224.73 ± 82.12	204.35 ± 82.66^∗^	236.6 ± 94.40	213.59 ± 88.50^∗^	−30.18, 13.59^a^ −28.48, 14.68^b^ −20.02, 22.81^c^	0.015
Arm (a.u.)	200.36 ± 47.75	191.03 ± 57.54		196.14 ± 44.15	173.70 ± 44.34^∗^	215.86 ± 57.51	190.26 ± 55.97^∗^	−11.34, 38.29^a^ −11.86, 38.12^b^ −25.97, 24.27^c^	0.034
Leg (a.u.)	234.43 ± 65.70	217.66 ± 72.61^∗^		245.75 ± 74.49	211.89 ± 67.75^∗^	246.98 ± 75.66	217.77 ± 74.28^∗^	−1.78, 34.41^a^ −6.16, 29.32^b^ −22.43, 12.96^c^	0.072

Skin oiliness									
Neck (a.u.)	10.74 ± 5.93	14.92 ± 13.74	15.00 ± 10.62		47.00 ± 41.51^∗^, ^#^	10.41 ± 6.71	40.02 ± 26.69^∗^, ^#^	−59.59, −3.35^a^ −52.02, −0.69^b^ −20.82, 32.42^c^	0.161
Back (a.u.)	4.94 ± 2.99	8.48 ± 5.32	6.14 ± 5.32		20.98 ± 18.18^∗^, ^#^	6.34 ± 5.17	15.35 ± 12.52^∗^	−28.68, −3.28^a^ −19.49, 4.97^b^ −3.23, 20.67^c^	0.176
Arm (a.u.)	4.98 ± 3.02	5.93 ± 4.77	10.31 ± 10.01		27.51 ± 20.37^∗^, ^#^	10.83 ± 14.19	36.12 ± 33.54^∗^, ^#^	−41.56, −2.30^a^ −45.16, −7.60^b^ −22.42, 13.52^c^	0.218
Leg (a.u.)	3.98 ± 2.78	6.08 ± 4.27	6.23 ± 4.78		18.97 ± 12.82^∗^, ^#^	7.05 ± 7.35	24.23 ± 17.24^∗^, ^#^	−24.68, −3.75^a^ −32.08, −5.35^b^ −20.72, 4.22^c^	0.226

Skin erythema									
Neck (a.u.)	365.66 ± 65.60	343.93 ± 72.78	369.41 ± 69.38		336.48 ± 75.47	367.52 ± 74.28	341.57 ± 72.57	−22.01, 43.19^a^ −28.02, 35.85^b^ −38.61, 25.26^c^	0.010
Back (a.u.)	212.98 ± 55.09	232.51 ± 60.37	230.46 ± 56.44		239.84 ± 68.42	236.23 ± 69.41	250.64 ± 68.34	−19.20, 35.69^a^ −24.46, 29.63^b^ −32.38, 21.06^c^	0.008
Arm (a.u.)	218.80 ± 44.48	223.86 ± 59.25	219.46 ± 52.15		211.66 ± 43.84	232.51 ± 55.25	227.18 ± 55.47	−14.17, 39.55^a^ −19.44, 33.54^b^ −32.11, 20.83^c^	0.020
Leg (a.u.)	207.34 ± 58.86	204.45 ± 66.69	217.37 ± 62.99		193.89 ± 59.12	210.96 ± 58.75	203.91 ± 60.39	−2.08, 41.37^a^ −17.42, 25.06^b^ −37.08, 5.43^c^	0.007

*Note:* Data are mean ± SD; *η* ^2^, eta squared.

Abbreviations: a.u., arbitrary unit; CI, confidence intervals.

^a^control group vs. Treatment‐1 group.

^b^control group vs. Treatment‐2 group.

^c^Treatment‐1 group vs. Treatment‐2 group.

^∗^significantly different from preintervention (*p* < 0.05).

^#^significantly different from the control group (*p* < 0.05).

### 3.3. Skin Moisture

**FIGURE 3:**
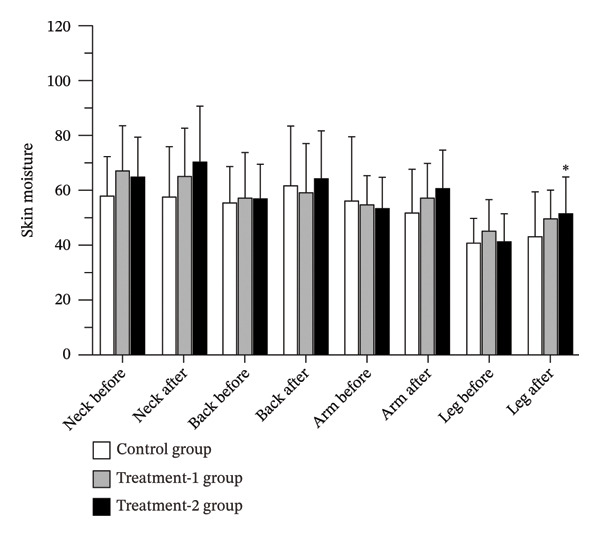
Skin moisture levels of participants before and after the 12‐week interventions.

Skin moisture levels remained unchanged in both the control and Treatment‐1 groups. In contrast, a significant increase in skin moisture was observed in the Treatment‐2 group at the leg region (*p* = 0.003). Additionally, there were trends toward increased moisture at the arm and back regions in the Treatment‐2 group (*p* = 0.096 and *p* = 0.093, respectively). Although no statistically significant differences were detected among groups overall, the Treatment‐2 group showed a tendency toward higher moisture levels at the neck region compared to the control group (*p* = 0.062) (Figure 3 and Table 2).

### 3.4. Skin Melanin

**FIGURE 4:**
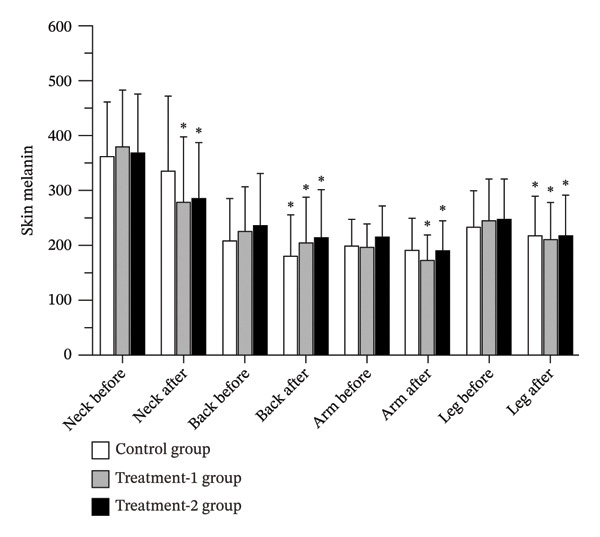
Skin melanin levels of participants before and after the 12‐week interventions.

The control group demonstrated a significant reduction in skin melanin levels at the back (*p* = 0.002) and leg (*p* = 0.01) regions. In contrast, both the Treatment‐1 group and the Treatment‐2 group exhibited significant decreases in melanin levels across all measured regions—neck, back, arm, and leg (*p* < 0.01). Despite these within‐group improvements, no statistically significant differences in melanin reduction were observed between the groups at any region (Figure 4 and Table 2).

### 3.5. Skin Oiliness

**FIGURE 5:**
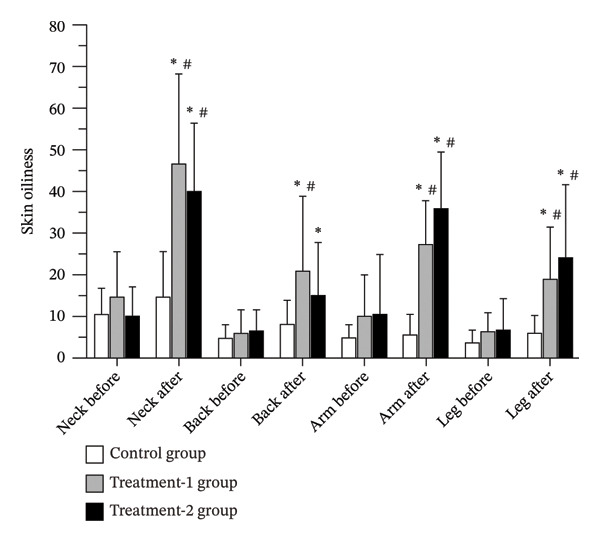
Skin oiliness levels of participants before and after the 12‐week interventions.

No significant change in skin oiliness was observed in the control group. In contrast, both the Treatment‐1 group and the Treatment‐2 group demonstrated significant increases in skin oiliness at the neck, back, arm, and leg regions (*p* < 0.05). Between‐group comparisons revealed that both treatment groups had significantly higher skin oiliness than the control group at the neck (Treatment‐1: *p* = 0.008; Treatment‐2: *p* = 0.048), arm (Treatment‐1: *p* = 0.032; Treatment‐2: *p* = 0.001), and leg (Treatment‐1: *p* = 0.017; Treatment‐2: *p* < 0.001). At the back region, a significant increase was observed only in the Treatment‐1 group compared to control (*p* = 0.025) (Figure 5 and Table 2).

### 3.6. Skin Erythema

**FIGURE 6:**
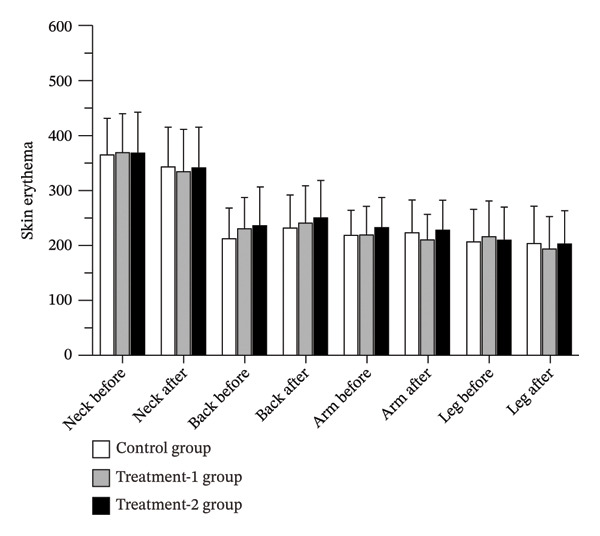
Skin erythema levels of participants before and after the 12‐week interventions.

Skin erythema levels were assessed to evaluate the safety and potential for irritation following 12 weeks of dermal application of coconut oil and snake fruit extract–infused oil. The results showed no significant changes in skin erythema within any of the groups postintervention (*p* > 0.05). Furthermore, no significant differences in erythema levels were observed among the three groups (*p* > 0.05), indicating that both oils were well tolerated and did not cause skin irritation (Figure 6 and Table 2).

## 4. Discussion

In this study, TTM was employed as the standardized intervention. TTM is a structured therapeutic technique combining deep tissue massage with sustained pressure along the ten principal energy lines, followed by passive stretching. Traditionally believed to promote energy flow and vitality [15], TTM is widely practiced in Thai traditional medicine [16] and increasingly recognized as a nonpharmacological intervention for musculoskeletal disorders such as myofascial pain syndrome, scapulocostal syndrome, and chronic back and neck pain [17].

Building upon this established modality and the documented antioxidant properties of snake fruit, we developed a massage oil infused with *S. zalacca* extract and applied it in combination with TTM to evaluate its effects on body skin quality. Participants receiving TTM with snake fruit extract–infused oil (Treatment‐2) demonstrated specific improvements, particularly in skin oiliness and localized moisture.

Importantly, all three groups exhibited significant improvements in skin elasticity across the neck, back, arm, and leg regions after 12 weeks, with no significant between‐group differences. These findings suggest that elasticity enhancement was largely attributable to the mechanical effects of massage itself. This interpretation aligns with previous reports. Caberlotto et al. [18] demonstrated improved skin structure and elasticity following 8 weeks of antiaging massage device use, associated with increased expression of decorin, fibrillin, tropoelastin, and procollagen‐1. Similarly, Cho et al. [19] reported improved elasticity in hypertrophic scar patients after prolonged massage therapy. Although the Treatment‐2 group showed numerically greater relative improvements (99% vs. 93% and 88%), these differences did not reach statistical significance, suggesting a limited additive effect beyond massage alone.

GC‐MS analysis identified dilauryl thiodipropionate and 2,5‐furandione as major constituents. Dilauryl thiodipropionate is a recognized antioxidant used in cosmetic formulations [20], while 2,5‐furandione has documented bioactive and antioxidant properties [21]. Antioxidants may support extracellular matrix integrity by mitigating oxidative damage, consistent with literature linking antioxidant supplementation to improved skin elasticity [22, 23]. However, given the absence of between‐group statistical differences, their contribution should be interpreted cautiously.

A significant increase in skin moisture was observed only in the Treatment‐2 group and specifically at the lower legs. Prior studies support the hydrating potential of botanical extracts. Meer et al. [13] reported improved hydration and elasticity after topical *Phoenix dactylifera* extract application. Plyduang et al. [24] demonstrated improved hydration and reduced transepidermal water loss (TEWL) following *Elaeis guineensis* extract use. Similar moisturizing effects have been reported for *Clitoria ternatea* and *Hylocereus polyrhizus* extracts [25], as well as *Carissa carandas* emulgel [26]. However, not all botanical formulations produce consistent hydration effects, as shown by Akhtar et al. [27]. Beyond the extract, the massage oil formulation itself contained mineral oil, jojoba oil, isopropyl myristate, alpha‐bisabolol, and tocopheryl acetate—ingredients known to enhance hydration and barrier function [28–30]. The region‐specific hydration effect, significant only at the lower legs, requires cautious interpretation. The lower extremities are physiologically more prone to dryness due to lower sebaceous gland density, reduced surface lipids, thicker stratum corneum, and greater TEWL. These characteristics may render them more responsive to occlusive or barrier‐enhancing interventions. In contrast, regions with higher baseline hydration (neck, back, arms) may exhibit smaller, nonsignificant changes. Nonetheless, the absence of baseline‐adjusted regional comparisons and correction for multiple site testing limits definitive interpretation. Therefore, the hydration effect should be considered preliminary and localized rather than generalized.

Reductions in the melanin intensity were observed across all groups, including the control group, suggesting that massage itself may influence pigmentation. Mechanical stimulation may enhance circulation and epidermal turnover [31] and modulate inflammatory and oxidative pathways involved in melanogenesis [32]. Previous studies show inconsistent findings: Cho et al. [19] reported melanin improvement following massage, whereas Nedelec et al. [33] observed no significant change. In the present study, greater reductions were observed in the oil‐treated groups. Fatty acids in coconut oil and snake fruit extract may influence tyrosinase degradation pathways [34, 35], while antioxidant constituents may inhibit melanogenesis [36]. However, seasonal variation or reduced UV exposure during follow‐up may also have contributed.

Skin oiliness increased substantially in both oil‐treated groups, far exceeding changes in the control group. Coconut oil, rich in medium‐chain triglycerides and polyphenols [37], is known to enhance barrier lipids and hydration [38, 39]. The greater oiliness observed in Treatment‐2 likely reflects its formulation, which includes mineral oil (65%), C11–12 isoparaffin, isopropyl myristate, and caprylic/capric triglyceride. Mineral oil reduces TEWL by forming an occlusive barrier [40] and is widely used for its protective properties [41]. The combined emollient effects of these agents likely explain the enhanced oiliness and localized hydration.

A key methodological consideration is the difference in base oils between groups. Coconut oil primarily supports barrier repair through lipid integration but has weaker occlusive properties [42], whereas mineral oil provides stronger occlusion and sustained moisture retention [40]. Thus, formulation effects likely contributed to the greater hydration and oiliness observed in Treatment‐2, and in the absence of a vehicle‐matched control, extract‐specific effects cannot be conclusively isolated.

Finally, the low extract concentration (0.0028% w/w) warrants cautious interpretation. Although phenolic compounds may exert biological activity at low concentrations via antioxidant and signaling mechanisms [43, 44], this study did not include phytochemical standardization, bioavailability assessment, or dose–response analysis. Given the modest and localized effects observed, the findings should be interpreted as preliminary evidence of feasibility rather than definitive proof of concentration‐dependent efficacy.

### 4.1. Study Limitations

To the best of our knowledge, this study is the first to develop a body massage oil infused with snake fruit extract and evaluate its effects in combination with TTM on body skin quality. Nevertheless, several limitations should be acknowledged. First, although all massage sessions were performed by licensed Thai traditional medicine practitioners to ensure procedural consistency, the same practitioner did not always treat the same participant due to randomized attendance schedules. While this approach reflects real‐world practice, minor intertherapist variability cannot be excluded. Second, although certain internal and external factors were controlled—participants were instructed to avoid other massage therapies, body oils, exfoliating products, and showering within 4 hours prior to assessment—daily lifestyle behaviors that may influence skin condition (e.g., sun exposure, diet, stress, and physical activity) were not restricted in order to preserve ecological validity. These uncontrolled variables may have influenced skin‐related outcomes. Third, although statistically significant changes were observed in selected parameters, the overall magnitude of effect was modest. Moreover, the improvement in skin moisture was region‐specific, reaching significance only at the lower legs. Without baseline‐adjusted regional comparisons, effect size analyses, or correction for multiple anatomical site testing, the possibility of Type I error or site‐dependent variability cannot be excluded. Therefore, the clinical relevance and treatment consistency require confirmation in larger, adequately powered randomized controlled trials with longer follow‐up. Fourth, a key methodological limitation is the difference in carrier oil composition between groups. Treatment‐1 used pure coconut oil, whereas Treatment‐2 incorporated snake fruit extract within a mineral oil–based carrier containing established occlusive agents. Because mineral oil reduces TEWL and enhances moisture retention, formulation‐related effects likely contributed to the observed increases in skin oiliness and localized hydration. In the absence of a vehicle‐matched control (i.e., the identical mineral oil base without extract), the independent contribution of snake fruit extract cannot be conclusively isolated. Fifth, mechanistic biomarkers—such as TEWL, stratum corneum lipid profiling, or inflammatory mediators—were not assessed, limiting the direct evaluation of barrier function and underlying biological mechanisms. Sixth, the final extract concentration (0.0028% w/w) was not evaluated in a dose–response design. Although botanical phytochemicals may exert activity at low concentrations, the absence of concentration‐ranging analysis, percutaneous absorption assessment, and phytochemical standardization limits definitive conclusions regarding extract‐specific potency. Finally, this trial was registered retrospectively. Although the protocol and outcome measures were finalized and approved by the institutional ethics committee prior to participant recruitment, delayed registration may limit transparency. Future trials will be prospectively registered in accordance with international best practices.

## 5. Conclusion

In conclusion, TTM resulted in significant improvements in skin elasticity and reductions in melanin levels across all study groups, indicating a strong effect of massage itself on these parameters. The application of massage oils—particularly snake fruit extract–infused oil—was associated with significantly greater increases in skin oiliness and a localized improvement in skin moisture at the leg region. These findings suggest that while snake fruit extract–infused oil does not confer superior effects on all skin parameters, it may provide specific benefits related to skin hydration and surface lipid content when used as an adjunct to TTM.

## Author Contributions

Piyapong Prasertsri conceived, designed, and planned the study. Piyapong Prasertsri, Suwipa Intakhiao, Warumpa Suwannarat, Thirapit Subongkot, Thanchanok Sirirak, Phannapat Piyaneeranat, and Sukrisd Koowattanatianchai implemented the study. Piyapong Prasertsri performed the data analysis. Piyapong Prasertsri and Suwipa Intakhiao drafted the manuscript.

## Funding

This research was funded by Burapha University (BUU); Thailand Science Research and Innovation (TSRI); and the National Science Research and Innovation Fund (NSRF) under the Fundamental Fund 2023, Grant No.: 31/2566.

## Disclosure

All authors reviewed and approved the final version of the manuscript.

## Conflicts of Interest

The authors declare no conflicts of interest.

## Supporting Information

CONSORT 2025 checklist.

## Supporting information


**Supporting Information** Additional supporting information can be found online in the Supporting Information section.

## Data Availability

The data are available upon request from the corresponding author.
